# Supplementary material for: Pneumococcal extracellular vesicles mediate horizontal gene transfer via the transformation machinery
Source: mSphere. 2024 Nov 6;9(12):e00727-24. doi: 10.1128/msphere.00727-24 (PMC11656791; doi:10.1128/msphere.00727-24)
Supplement: Fig. S1 — Representative NTA and additional pEV cryoEM images. [file msphere.00727-24-s0001.docx]

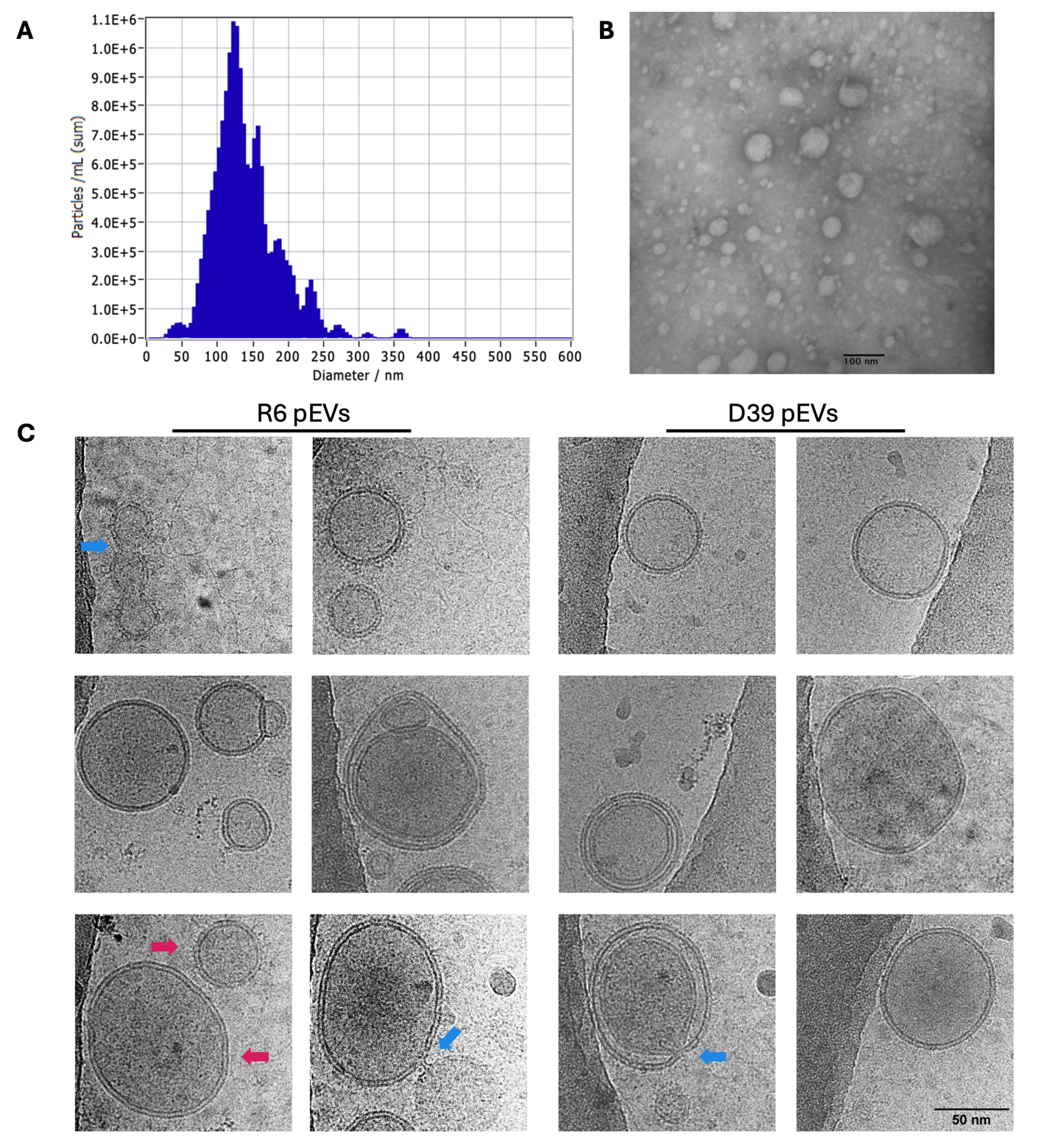


**SFig.1**: (**A**) Representative nanoparticle tracking analysis of pneumococcal extracellular vesicles isolated from R6 (produced by the software ZetaView, using 1:1000 dilution of EV fraction). (**B**) Representative negative strain electron micrograph of pEVs from R6, scale bar 100 nm. (**C**) Images selected from cryo-electron micrographs of pEVs from R6 and D39. Magenta arrows indicate two pEVs representing either smooth or textured surfaces. Blue arrows indicate apparent fusion or fission events (rare in our set). Doublet and triplet pEVs are also relatively rare in our set. All images are the same scale (scale bar of 50nm in final image).
