# Supplementary material for: Pneumococcal extracellular vesicles mediate horizontal gene transfer via the transformation machinery
Source: mSphere. 2024 Nov 6;9(12):e00727-24. doi: 10.1128/msphere.00727-24 (PMC11656791; doi:10.1128/msphere.00727-24)
Supplement: Fig. S2 — pEV particles follow Brownian trajectories. [file msphere.00727-24-s0002.docx]

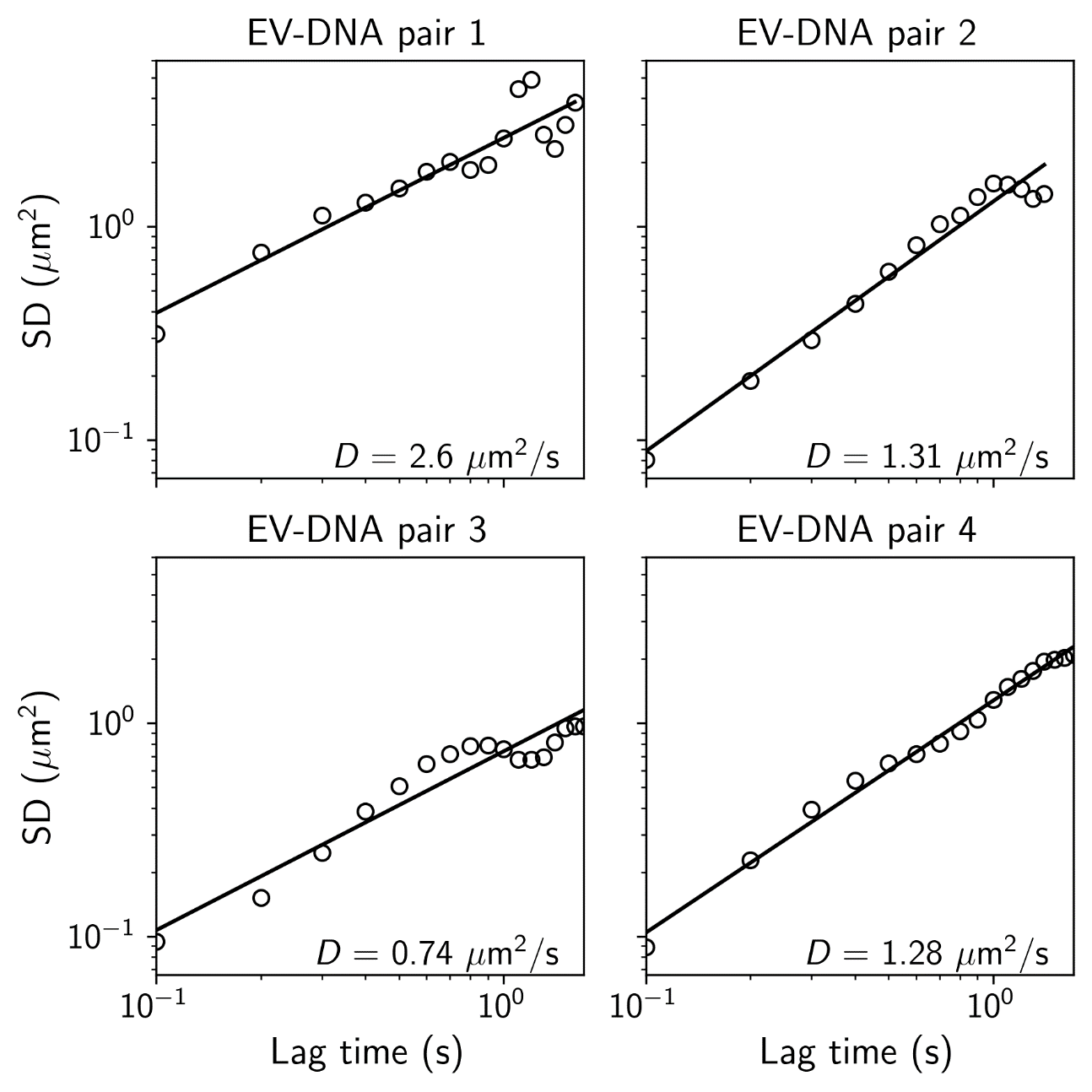


**SFig.2**: pEV particles follow Brownian trajectories. Points represent computed squared displacements (SD) as a function of lag time for tracked pEV particles. Lines represent linear fits to the data in log-space for each pEV particle. D = Diffusion coefficients.
