# Supplementary material for: Pneumococcal extracellular vesicles mediate horizontal gene transfer via the transformation machinery
Source: mSphere. 2024 Nov 6;9(12):e00727-24. doi: 10.1128/msphere.00727-24 (PMC11656791; doi:10.1128/msphere.00727-24)
Supplement: Fig. S3 — PCR amplification and DNA quantification. [file msphere.00727-24-s0003.docx]

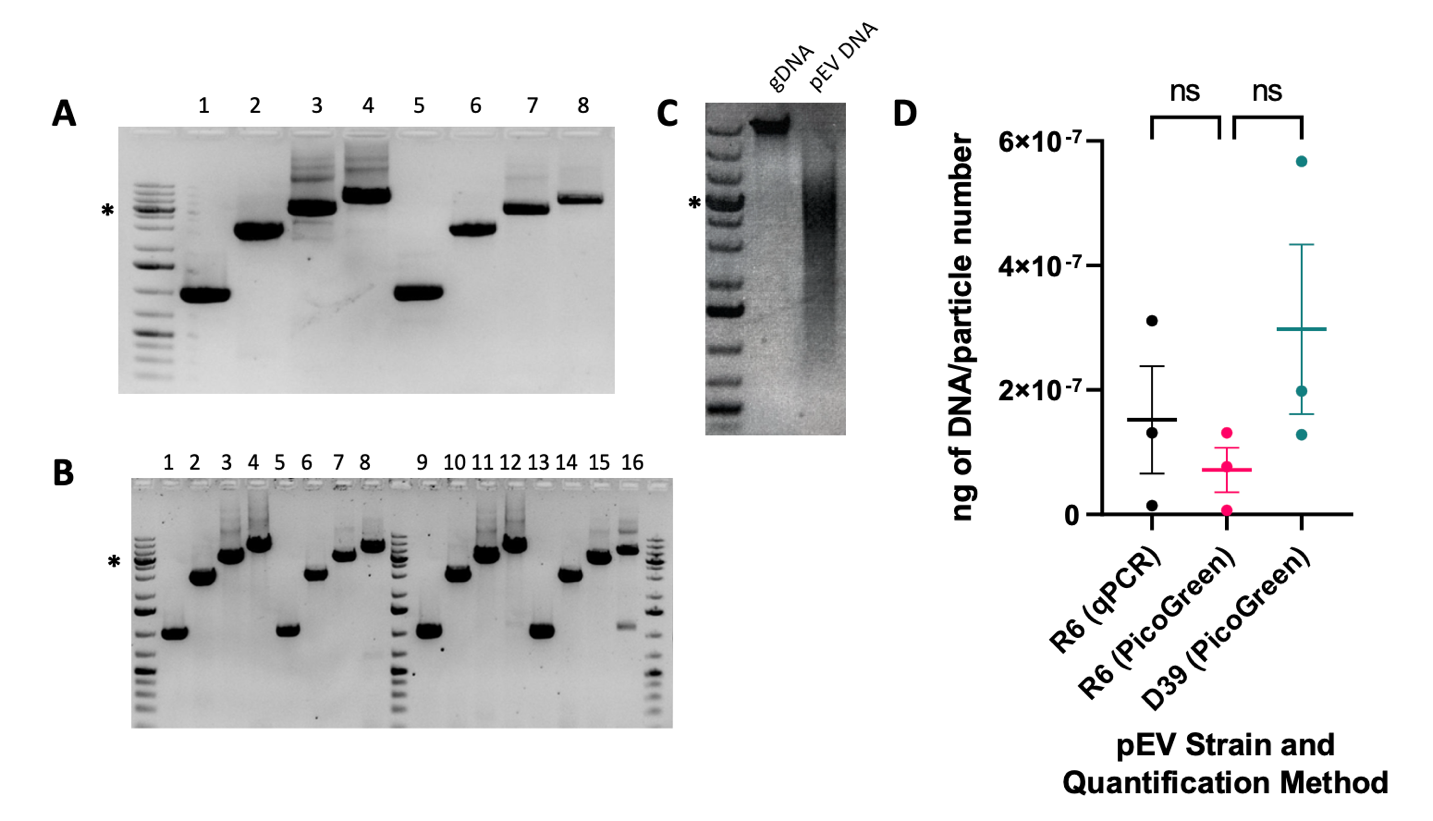


**SFig.3**: **(A-B)** PCR amplification of 1, 3, 5, and 7 kb DNA fragments from R6 pEVs and genomic DNA. **(A)** PCR primers targeted to gene spr1608. Left lane is the MW standard; lanes labeled 1-4 are positive control where genomic DNA was used as template, and lanes 5-8 used pEVs as template. PCR was performed on two independent sets of vesicles. **(B)** PCR primers targeted to gene spr0001 (left half of the gel) and spr0065 (right half of the gel). Lanes 1-4 and 9-12 are positive control where genomic DNA was used as template, lanes 5-8 and 13-18 used pEVs as template. **(C)** Gel electrophoresis profile of DNA purified from bacterial culture (gDNA) or SEC-purified pEVs (pEV DNA). Asterisk (*) indicates 5,000 base pair marker on the GeneRuler 1 kb Plus DNA Ladder (Invitrogen). **(D)** Quantification of pEV DNA by qPCR and PicoGreen. qPCR was performed on three R6 pEV samples and each point represents four technical replicates. PicoGreen staining was performed on the same three R6 pEV samples and three D39 pEV samples. These data are normalized to the number of pEVs in each sample as measured by NTA. One-way ANOVA compared to R6 (PicoGreen), ns=p>0.5.
