# Supplementary material for: Pneumococcal extracellular vesicles mediate horizontal gene transfer via the transformation machinery
Source: mSphere. 2024 Nov 6;9(12):e00727-24. doi: 10.1128/msphere.00727-24 (PMC11656791; doi:10.1128/msphere.00727-24)
Supplement: Fig. S4 — pEVs mediate horizontal gene transfer. [file msphere.00727-24-s0004.docx]

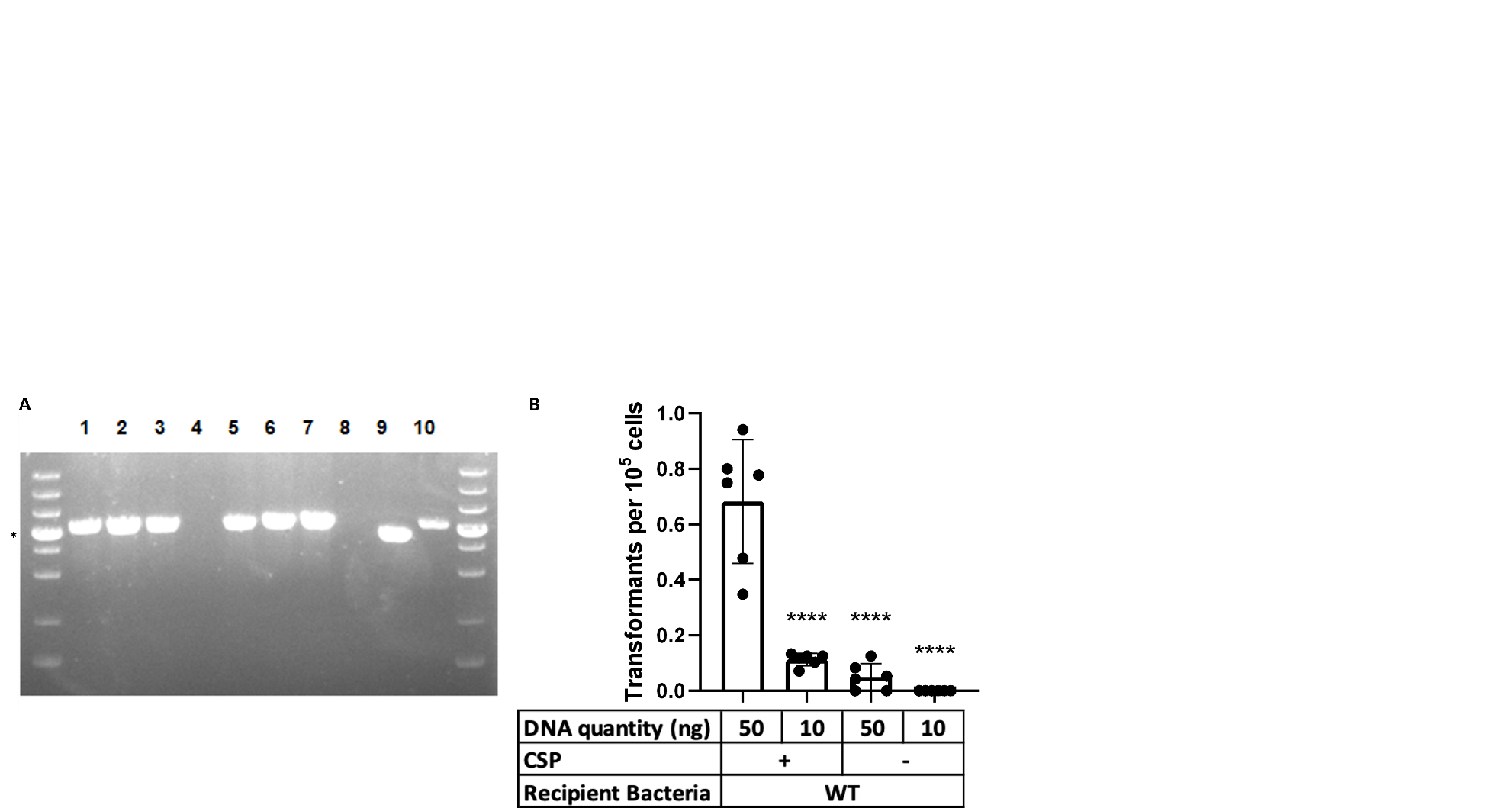


**SFig.4: pEVs mediate horizontal gene transfer**. **(A)** Three colonies from the transformation plates were grown in rich media overnight and used as a PCR template to check for presence of genes encoding spectinomycin resistance. Every colony produced amplicons of the appropriate size. The templates are as follows: Lanes 1-3 and 5-7: transformation colonies; lane 4: growth media; lane 8 no template; lane 9: gDNA from wild-type R6 SpecS; and lane 10: genomic data from donor bacteria R6-SpecR. Asterisk (*) indicates 5,000 base pair marker on the GeneRuler 1 kb Plus DNA Ladder (Invitrogen). **(B)** D39 cells (SpecS background) were exposed to pEV DNA from a D39-SpecR strain. Transformations were performed with and without CSP. Bars represent mean + SEM with dots overlayed within a bar representing a data point from each independent experiment (n=6, **** adjusted p-value < 0.0001 for Dunnett’s multiple comparison test).
