# Supplementary material for: Pneumococcal extracellular vesicles mediate horizontal gene transfer via the transformation machinery
Source: mSphere. 2024 Nov 6;9(12):e00727-24. doi: 10.1128/msphere.00727-24 (PMC11656791; doi:10.1128/msphere.00727-24)
Supplement: Supplemental Material — Legends for supplemental video, figures, and tables. [file msphere.00727-24-s0005.docx]

**Legends for Supplemental Material**

**SVideo.1**: Co-localization and co-diffusion of pEV and DNA particles. DNA particles are false colored in green (left panel), pEV particles are false colored in magenta (middle panel), and the merge is present in the right panel. pEV sample was treated with PicoGreen and DiD to label DNA and the pEV membrane, respectively. Scale bar is indicated.

**SFig.1**: (**A**) Representative nanoparticle tracking analysis of pneumococcal extracellular vesicles isolated from R6 (produced by the software ZetaView, using 1:1000 dilution of EV fraction). (**B**) Representative negative strain electron micrograph of pEVs from R6, scale bar 100 nm. (**C**) Images selected from cryo-electron micrographs of pEVs from R6 and D39. Magenta arrows indicate two pEVs representing either smooth or textured surfaces. Blue arrows indicate apparent fusion or fission events (rare in our set). Doublet and triplet pEVs are also relatively rare in our set. All images are the same scale (scale bar of 50nm in final image).

**SFig.2**: pEV particles follow Brownian trajectories. Points represent computed squared displacements (SD) as a function of lag time for tracked pEV particles. Lines represent linear fits to the data in log-space for each pEV particle. D = Diffusion coefficients.

**SFig.3**: **(A-B)** PCR amplification of 1, 3, 5, and 7 kb DNA fragments from R6 pEVs and genomic DNA. **(A)** PCR primers targeted to gene spr1608. Left lane is the MW standard; lanes labeled 1-4 are positive control where genomic DNA was used as template, and lanes 5-8 used pEVs as template. PCR was performed on two independent sets of vesicles. **(B)** PCR primers targeted to gene spr0001 (left half of the gel) and spr0065 (right half of the gel). Lanes 1-4 and 9-12 are positive control where genomic DNA was used as template, lanes 5-8 and 13-18 used pEVs as template. **(C)** Gel electrophoresis profile of DNA purified from bacterial culture (gDNA) or SEC-purified pEVs (pEV DNA). Asterisk (*) indicates 5,000 base pair marker on the GeneRuler 1 kb Plus DNA Ladder (Invitrogen). **(D)** Quantification of pEV DNA by qPCR and PicoGreen. qPCR was performed on three R6 pEV samples and each point represents four technical replicates. PicoGreen staining was performed on the same three R6 pEV samples and three D39 pEV samples. These data are normalized to the number of pEVs in each sample as measured by NTA. One-way ANOVA compared to R6 (PicoGreen), ns=p>0.5.

**SFig.4: pEVs mediate horizontal gene transfer**. **(A)** Three colonies from the transformation plates were grown in rich media overnight and used as a PCR template to check for presence of genes encoding spectinomycin resistance. Every colony produced amplicons of the appropriate size. The templates are as follows: Lanes 1-3 and 5-7: transformation colonies; lane 4: growth media; lane 8 no template; lane 9: gDNA from wild-type R6 SpecS; and lane 10: genomic data from donor bacteria R6-SpecR. Asterisk (*) indicates 5,000 base pair marker on the GeneRuler 1 kb Plus DNA Ladder (Invitrogen). **(B)** D39 cells (SpecS background) were exposed to pEV DNA from a D39-SpecR strain. Transformations were performed with and without CSP. Bars represent mean + SEM with dots overlayed within a bar representing a data point from each independent experiment (n=6, **** adjusted p-value < 0.0001 for Dunnett’s multiple comparison test).

**Table S1.** Characterization via Nanoparticle Tracking Analysis. Table displays the EV size in nanometers and concentration in particles/ml. Values represent three biological replicates of R6 EVs, each one with at least 3 technical replicates. Technical replicates were averaged to generate a value per experiment, the median was calculated for the 3 experiments.

**Table S2.** Primers for pneumococcal regions.

**Table S3.** Gibson assembly primers and sequence check primers.
