# Supplementary material for: Pneumococcal extracellular vesicles mediate horizontal gene transfer via the transformation machinery
Source: mSphere. 2024 Nov 6;9(12):e00727-24. doi: 10.1128/msphere.00727-24 (PMC11656791; doi:10.1128/msphere.00727-24)
Supplement: Supplemental tables — Tables S1 to S3. [file msphere.00727-24-s0006.docx]

**Table S1. Characterization via Nanoparticle Tracking Analysis.** Table displays the EV size in nanometers and concentration in particles/ml. Values represent three biological replicates of R6 EVs, each one with at least 3 technical replicates. Technical replicates were averaged to generate a value per experiment, the median was calculated for the 3 experiments.

| Biological replicate | Size (nm) | Particles/ml |
| --- | --- | --- |
| 1 | 133.3 | 2.2E+10 |
| 1 | 129.4 | 3.3E+10 |
| 1 | 128.4 | 2.8E+10 |
| 1 | 128.9 | 3.2E+10 |
| 2 | 131.1 | 2.3E+10 |
| 2 | 132.3 | 2.7E+10 |
| 2 | 131.8 | 2.4E+10 |
| 3 | 129.5 | 1.7E+10 |
| 3 | 127.1 | 1.8E+10 |
| 3 | 124.1 | 1.7E+10 |
| 3 | 120.6 | 2.6E+10 |
| **Median** | 130.0 | 2.4E+10 |

**Table S2. Primers for pneumococcal regions.**

| Target gene | Primer Name | Sequence |
| --- | --- | --- |
| spr.0001 | R6.peg.1 F | AGGTAGAGGAAAATGTTGCCA |
|  | R6.peg.1 1026 bp R | CACCATAAAAGTTACCAACTTCAGT |
|  | R6.peg.1 3000 bp R | AATGGGTAGTTTGCTGCCTC |
|  | R6.peg.1 5000 bp R | CCCTTTCTTAGATGAATCAGTCAA |
|  | R6.peg.1 7000 bp R | ATTCCCAGCATAGACATATGGA |
| spr.0065 | R6.peg.65 F | ATGAAAGCATACACAGAGCGTG |
|  | R6.peg.65 1000 bp R | AGGTTTGACCAGCTTTAAGAATG |
|  | R6.peg.65 3000 bp R | AGGTGGTATGAATACTTGGCCC |
|  | R6.peg.65 5000 bp R | GTGAGCTTTCAACTTGGACAAA |
|  | R6.peg.65 7000 bp R | AGCCTTTGAAGAACTTTTACTAGAAGAC |
| spr.1412 | R6.peg.1411 F | GCTCGCTTTGAAGATAGGTTT |
|  | R6.peg.1411 1050 bp R | CCTGCACCAAGAGATTAAAATC |
|  | R6.peg.1411 3053 bp R | TAATCGGGATTCGGATGG |
|  | R6.peg.1411 5029 bp R | TAATCTTATAAGCACCTGCAAGG |
|  | R6.peg.1411 5000 bp R | GCCATCCGAAGATGACTTTT |
| spr.1825 | spGAPDH F | GGTCGTCTTGCTTTCCGTCGTATC |
|  | spGAPDH R | GCTTTCATAGCTGCGTTCACTTCATC |
| spr.1608 | R6.peg.2149 F | ATGAGATACATAACTCTTGGTCAAGATG |
|  | R6.peg.2149 1018 bp R | CCTTTAATGTCAATAATTCTCCCC |
|  | R6.peg.2149 3004 bp R | GTTCGTTTGGTCTAGTTGCAAC |
|  | R6.peg.2149 5000 bp R | AAAATGGCTCTCGAACTGAGTA |
|  | R6.peg.2149 7000 bp R | AATACATGAGCAGGAAGGATAACA |

**Table S3. Gibson assembly primers and sequence check primers.**

| Construct | Primer Name | Sequence |
| --- | --- | --- |
| 1900 Spec | 14381​ F1_fwd | CGTGAAGCTATCCAAGAAGAAGC |
|  | 14382​ F1_rev | ATATATGGATCCCTGCTCCTAGGGAGATTTATCTTT |
|  | 14383​ F2_fwd | ATATATCCCGGGGTCGCTCCGTCGTTCGATTTCCTA |
|  | 14384​ F2_rev | CCATCTCAGAGATATGAACAAGGGCATCTGTC |
|  | 14385​ 1900specCassette_fwd | TTACCGTAAAGGTGAATTGT |
|  | 14386​ 1900specCassette_rev | TAAACTTCATCCACTTTGGC |
|  | 13021 Spec_fwd | ATATGGATCCTCCCCCGTTTGATTTTTAATGGTAA  TGTGATAAA |
|  | 13022 Spec_rev | ATATCCCGGG CGGAATGGATCCAATTTTTTTA |
| *ΔcomEA/comEC* | comEA/EC KO F1_fwd | GGTACACCACCAAGTAGAAC |
|  | comEA/EC KO F1_rev | TAGGCATAGACTCGATAATTGCTTCCATATTTTC |
|  | comEA/EC KO Kan Term_fwd | AATTATCGAGTCTATGCCTATTCCAGAGGAAATGG |
|  | comEA/EC KO Kan term_rev | TCGATTTTCCGCGAAAAAACCCCGCCGAAG |
|  | comEA/EC KO F2_fwd | GTTTTTTCGCGGAAAATCGAAAGT GTTCG |
|  | comEA/EC KO F2_rev | CCAAGCTGACTGAGTTTG |
| 1900 Spec | Spec_seq_Up | GGA TGA TTC CAC GGT ACC |
| 1900 Spec | Spec_seq_Down | GGG AGA GAA TTT TGT TAG CAG TT |
| *ΔcomEA/comEC* | Kan_seq_Up | CCACCAGCTTATATACCTTAGC |
| *ΔcomEA/comEC* | Kan_seq_Down | GGACAAGTGGTATGACATTGC |
